# Co-occurring pathogenic variants in 6q27 associated with dementia spectrum disorders in a Peruvian family

**DOI:** 10.3389/fnmol.2023.1104585

**Published:** 2023-02-16

**Authors:** Karla Lucia F. Alvarez, Jorge Alberto Aguilar-Pineda, Michelle M. Ortiz-Manrique, Marluve F. Paredes-Calderon, Bryan C. Cardenas-Quispe, Karin Jannet Vera-Lopez, Luis D. Goyzueta-Mamani, Miguel Angel Chavez-Fumagalli, Gonzalo Davila-Del-Carpio, Antero Peralta-Mestas, Patricia L. Musolino, Christian Lacks Lino Cardenas

**Affiliations:** ^1^Laboratory of Genomics and Neurovascular Diseases, Universidad Católica de Santa María, Arequipa, Peru; ^2^Division of Neurology, Psychiatry and Radiology of the National Hospital ESSALUD-HNCASE, Arequipa, Peru; ^3^Vicerrectorado de Investigación, Universidad Católica de Santa María, Arequipa, Peru; ^4^Department of Neurology, Massachusetts General Hospital, Boston, MA, United States; ^5^Center for Genomic Medicine, Massachusetts General Hospital, Boston, MA, United States; ^6^Cardiovascular Research Center, Cardiology Division, Massachusetts General Hospital, Boston, MA, United States

**Keywords:** unbiased gene discovery, whole genome sequencing, family-specific genetic factor, Amerindian ancestral background, Alzheimer’s disease

## Abstract

Evidence suggests that there may be racial differences in risk factors associated with the development of Alzheimer’s disease and related dementia (ADRD). We used whole-genome sequencing analysis and identified a novel combination of three pathogenic variants in the heterozygous state (*UNC93A*: rs7739897 and *WDR27*: rs61740334; rs3800544) in a Peruvian family with a strong clinical history of ADRD. Notably, the combination of these variants was present in two generations of affected individuals but absent in healthy members of the family. *In silico* and *in vitro* studies have provided insights into the pathogenicity of these variants. These studies predict that the loss of function of the mutant UNC93A and WDR27 proteins induced dramatic changes in the global transcriptomic signature of brain cells, including neurons, astrocytes, and especially pericytes and vascular smooth muscle cells, indicating that the combination of these three variants may affect the neurovascular unit. In addition, known key molecular pathways associated with dementia spectrum disorders were enriched in brain cells with low levels of UNC93A and WDR27. Our findings have thus identified a genetic risk factor for familial dementia in a Peruvian family with an Amerindian ancestral background.

## 1. Introduction

Neurological disorders are an important cause of disability and death around the world. Interestingly, Alzheimer’s disease, dementia, Parkinson’s disease, epilepsy, schizophrenia, and autism spectrum disorder share common anatomical alterations and cognitive defects (Kochunov et al., 2021). Certainly, Alzheimer’s disease is the main cause of dementia, contributes 50 to 75% of cases (Report, 2021), and can be presented in two forms as defined by age: early-onset Alzheimer’s disease (EOAD), which occurs before 65 years of age, and late-onset Alzheimer’s disease (LOAD), which is mostly present after 65 years of age (Mendez, 2017). Both EOAD and LOAD have a family origin and involve an inheritance mode of autosomal dominant transmission (Gatz et al., 2006). Genetic variations in three genes, namely, amyloid precursor protein (APP), presenilin 1, and presenilin 2 (PSEN1 and PSEN2), are nearly 100% penetrant and were identified as causative of EOAD (Van Cauwenberghe et al., 2016). On the contrary, the expression of the apolipoprotein E (APOE) ε4 gene is the major risk factor for LOAD in the Caucasian population (Guerreiro et al., 2012). There has been evidence, however, that the risk of developing Alzheimer’s disease in ε4 carriers differs among ethnic groups. For instance, ε4 carriers of African descent have a low risk of Alzheimer’s disease (Farrer et al., 1997), while Amerindian genetic ancestry seemed to be protected from cognitive decline (Granot-Hershkovitz et al., 2021). Similarly, variants in the TREM2 (R47H, H157Y, and L211P) genes, which are closely associated with Alzheimer’s disease in the Caucasian population (Guerreiro et al., 2013; Li et al., 2021), were not replicated in Japanese descendants (Miyashita et al., 2014). These epidemiological observations indicate that genetic risk factors for neurological disorders have different effects between ethnicities.

In recent years, genome-wide association studies (GWAS) have permitted the identification and characterization of multiple genetic risk loci associated with Alzheimer’s disease and related dementia (ADRD; Kunkle et al., 2019). Most of these genetic loci were not associated with the APP processing, however, but rather with the immune response (TREM2, CLU, CR1, CD33, EPHA1, and MS4A4A/MS4A6E), endosomal trafficking (PICALM, BIN1, and CD2AP), or lipid metabolism (ABCA7; Bellenguez et al., 2020). An overlap between Alzheimer’s disease pathogenic variants and other neurogenerative or neuropsychiatric disorders has also been reported, indicating a shared genetic and molecular origin. For example, an Alzheimer’s disease variant in the TREM2 gene (rs75932628) was also correlated with amyotrophic lateral sclerosis (Cady et al., 2014), while a variant in the MARK2 gene (rs10792421) was associated with Alzheimer’s disease and bipolar disorder (Drange et al., 2019).

Identifying individuals at high risk of ADRD remains a global health need and a major challenge for minority populations. Here, we performed a whole-genome sequencing (WGS) analysis for a Peruvian family with a strong clinical history of ADRD, including Alzheimer’s disease and dementia. We also explored the effect of these variants on the neurovascular unit of the brain through *in silico* and *in vitro* studies.

## 2. Materials and methods

### 2.1. Patient sample collection

A family (*n* = 14) originally from Peru, with five members diagnosed with neurological and neuropsychiatry disorders, was enrolled in this study. Non-familial patients with Alzheimer’s disease (*n* = 8) and healthy individuals (*n* = 50) were recruited for the variant validation study. The selection criteria for the healthy individuals were as follows: age > 60 years, without signs of dementia, and no familial history of Alzheimer’s disease. Probable Alzheimer’s disease was diagnosed according to the guidelines of the National Institute of Neurological and Communicative Disorders and the Stroke and Alzheimer Disease and Related Disorders Association (McKhann et al., 2011). Whenever possible, the cognitive status of each family member was diagnosed based on a neuropsychological test (MoCA blind test and the clock drawing test). The cutoff for a normal MoCA score was 18, and for a normal clock drawing test was 6.

### 2.2. Genetic analysis

Genomic DNA was extracted from saliva samples using the prepIT.L2P reagent (Genotek, Cat. No PT-L2P-5) according to the manufacturer’s instructions. The qualifying genomic DNA samples were randomly fragmented using Covaris Technology, obtaining a fragment of 350 bp. The DNA nanoballs (DNBs) were produced using rolling circle amplification (RCA), and the qualified DNBs were loaded into the patterned nanoarrays. The WGS was conducted on the BGISEQ-500 platform (BGI Genomics, Shenzhen, China). Raw sequencing reads were aligned to the human reference genome (GRCh38/HG38) with the Burrows–Wheeler Aligner (BWA) software and variant calling was performed with the Genome Analysis Toolkit (GATK v3.5) according to best practice. On average, 88.10% mapped successfully and 93.23% mapped uniquely. The duplicate reads were removed from the total mapped reads, resulting in a duplicate rate of 2.48% and a 30.72-fold mean sequencing depth on the whole genome, excluding gap regions. On average per sequencing individual, 99.34% of the whole genome, excluding gap regions, were covered by at least 1 × coverage, 98.78% had at least 4 × coverage, and 97.42% had at least 10 × coverage. The whole-genome sequencing analysis pipeline is presented in Supplementary Figure 1.

### 2.3. Sanger sequencing

Based on the results of the WGS, the variants that were present in affected members of the family but absent in healthy individuals were validated using Sanger sequencing. All PCR products were sequenced using an ABI 3130 Genetic Analyzer. Sequence analysis was performed with the Chromas program in the DNASTAR analysis package. The PCR and sequencing primers are shown in Table 1.

**Table 1:** Polymerase chain reaction and sequencing primers.

Gene	SNP ID	PCR primers		Sequencing primers
		Forward primer sequence (5′–3′)	Reverse primer sequence (5′–3′)	Forward primer sequence (5′–3′)
WDR27	rs3800544	GTTTGCGCTCCTAGTTTCATG	GCATTCCGTACTTCCTTCCATC	TGTCCTACCGACCTCTCCACTG
WDR27	rs61740334	ACTGTGAATGTCTCCCGATCAC	ACTTGAAGTTGCATGGCATGG	TTCCCTCAGGGAGGCATAC
UNC93A	rsRS7739897	TACGGCGTTCTGTTTGAGAAG	TCAACCAGGCAGAGGATGAAG	GCTGCCTTGTCGCCAATTAC

### 2.4. Cell lines

Primary human vascular smooth muscle cells (VSMCs) from carotid of healthy donors were purchased from Cell Applications Inc. (Cat. No 3514k-05a, neural crest origin). Human brain vascular pericytes were purchased from ScienCell (Cat. No 1200). Human neurons (SH-SY5Y) were purchased from ATCC (Cat. No CRL-2266). Human astrocytes were purchased from Cell Applications Inc. (Cat. No 882A05f).

### 2.5. RNA sequencing and qPCR

Total RNA was extracted using a miRNeasy kit (Qiagen, Cat. No 217084) following the manufacturer’s protocol instructions. The BGISEQ platform was used for RNA-seq, generating some 4.28G Gb bases per sample, on average. The average mapping ratio with the reference genome was 97.01% and the average mapping ratio with the gene was 74.05%; 17,029 genes were identified. We used HISAT to align the clean reads to the reference genome and Bowtie2 to align the clean reads to the reference genes. A total of 100 ng of total RNA was used for qPCR as the starting template for cDNA synthesis. The cDNA was prepared by reverse transcription (RT), and gene expression was analyzed by quantitative PCR (qPCR) on an SYBR green system (Applied Biosystems). Expression results were analyzed using the DDCT method, and GAPDH (encoding glyceraldehyde-3-phosphate dehydrogenase) was used as a housekeeping gene. Fold changes were calculated as the average relative to the control carotid as the baseline.

### 2.6. Computational details

#### 2.6.1. System building, structural refinements, and molecular dynamic simulations (MDS)

The Q86WB7–1 (UNC93A, 457 aa) and A2RRH5-4 (WDR27, 827 aa) sequences (WDR27, 2020; Wang et al., 2021) were used to build the 3D wild-type protein structures using the I-TASSER server (Zheng et al., 2021). The mutant variants (UNC93A V409I, and WDR27 R467H-T542S) were built based on these 3D models by performing site-direct mutagenesis using UCSF Chimera software (Pettersen et al., 2004). To avoid the residue overlapping in all protein systems, a structural refinement was carried out using the ModRefiner server (Xu and Zhang, 2011). Classical MD simulations were performed using the GROMACS 2020.4 package with the OPLS-AA force field parameters (Jorgensen et al., 1996; Kutzner et al., 2019). All protein systems were built in a triclinic simulation box with periodic boundary conditions (PBC) in all directions (*x*, *y*, and *z*). They were then solvated using the TIP4P water model (Jorgensen et al., 1983), and Cl^–^ or Na^+^ ions were used to neutralize the total charge in the simulation box. The mimicking of physiological conditions was performed by ionic strengthening, with the addition of 150 mM NaCl. The distance of the protein surfaces to the edge of the periodic box was set at 1.5 nm, and a 1 fs step was applied to calculate the motion equations using the Leapfrog integrator (Hockney et al., 1974). The temperature for proteins and water-ions in all simulations was set at 309.65 K using the modified Berendsen thermostat (V-rescale algorithm), with a coupling constant of 0.1 ps (Berendsen et al., 1984). The pressure was maintained at 1 bar using the Parrinello–Rahman barostat with a compressibility of 4.5 × 10^−5^ bar^−1^ and coupling constant of 2.0 ps (Bussi et al., 2007). The particle mesh Ewald method was applied to long-range electrostatic interactions with a cutoff equal to 1.1 nm for nonbonded interactions, with a tolerance of 1×10^5^ for contribution in the real space of the 3D structures. The Verlet neighbor searching cutoff scheme was applied with a neighbor-list update frequency of 10 steps (20 fs). Bonds involving hydrogen atoms were constrained using the linear constraint solver (LINCS) algorithm (Hess, 2008). The energy was minimized in all simulations with the steepest descent algorithm for a maximum of 100,000 steps. We performed two steps for the equilibration process; one step of dynamics (1 ns) in the NVT (isothermal-isochoric) ensemble, followed by 2 ns of dynamics in the NPT (isothermal-isobaric) ensemble. The final simulation was then performed in the NPT ensemble for 500 ns followed by the analysis of the structures and their energy properties.

#### 2.6.2. Structural and energetic analysis of 3D protein structures

All MD trajectories were corrected, and the 3D structures were recentered in the simulation boxes. RMSD, RMSF, the radius of gyrations, H-bonds, residue distances, and solvent-accessible surface area analyses were performed using the Gromacs tools, and the results were plotted using XMGrace software. We used the UCSF Chimera, VMD software packages, to visualize the structures. Atomic interactions and 2D plots were analyzed using the LigPlot software packages (Wallace et al., 1995). Electrostatic potential (ESP) surfaces were calculated using the APBS (Adaptive Poisson-Boltzmann Solver) software, and the PDB2PQR software was used to assign the charges and radii to protein atoms (Baker et al., 2001).

#### 2.6.3. Calculation of binding free energy

The Molecular Mechanics Poisson–Boltzmann Surface Area (MM/PBSA) of free energies and energy contribution by individual residues was calculated to analyze the effect of amino-acid substitutions on the different structures using the last 100 ns of MD trajectories and the g_mmpbsa package (Kumari et al., 2014). The interacting energy was calculated using the following equation:


$$\Delta G_{\mathrm{int}}=G_{\mathrm{Prot}}-\left(G_{1}\right)$$

where the term *G*_1_ is the free energy of the different sites of the protein, and *G*_Prot_ is the free energy of the entire 3D structure. In this context, the free energy of each term was calculated as follows:


$$G_{x}=E_{\mathrm{MM}}+G_{\mathrm{solv}}-TS$$

where *E*_MM_ is the standard mechanical energy (MM) produced from bonded interactions, electrostatic interactions, and van der Waals interactions. *G*_solv_ is the solvation energy that includes the free energy contributions of the polar and nonpolar terms. The TS term refers to the entropic contribution and was not included in this calculation due to the computational costs (Rastelli et al., 2010; Kumari et al., 2014). Finally, 309 Kelvin (K) of temperature was used as the default parameter in all our calculations.

## 3. Results

### 3.1. Genetic analyses

**Figure 1:**
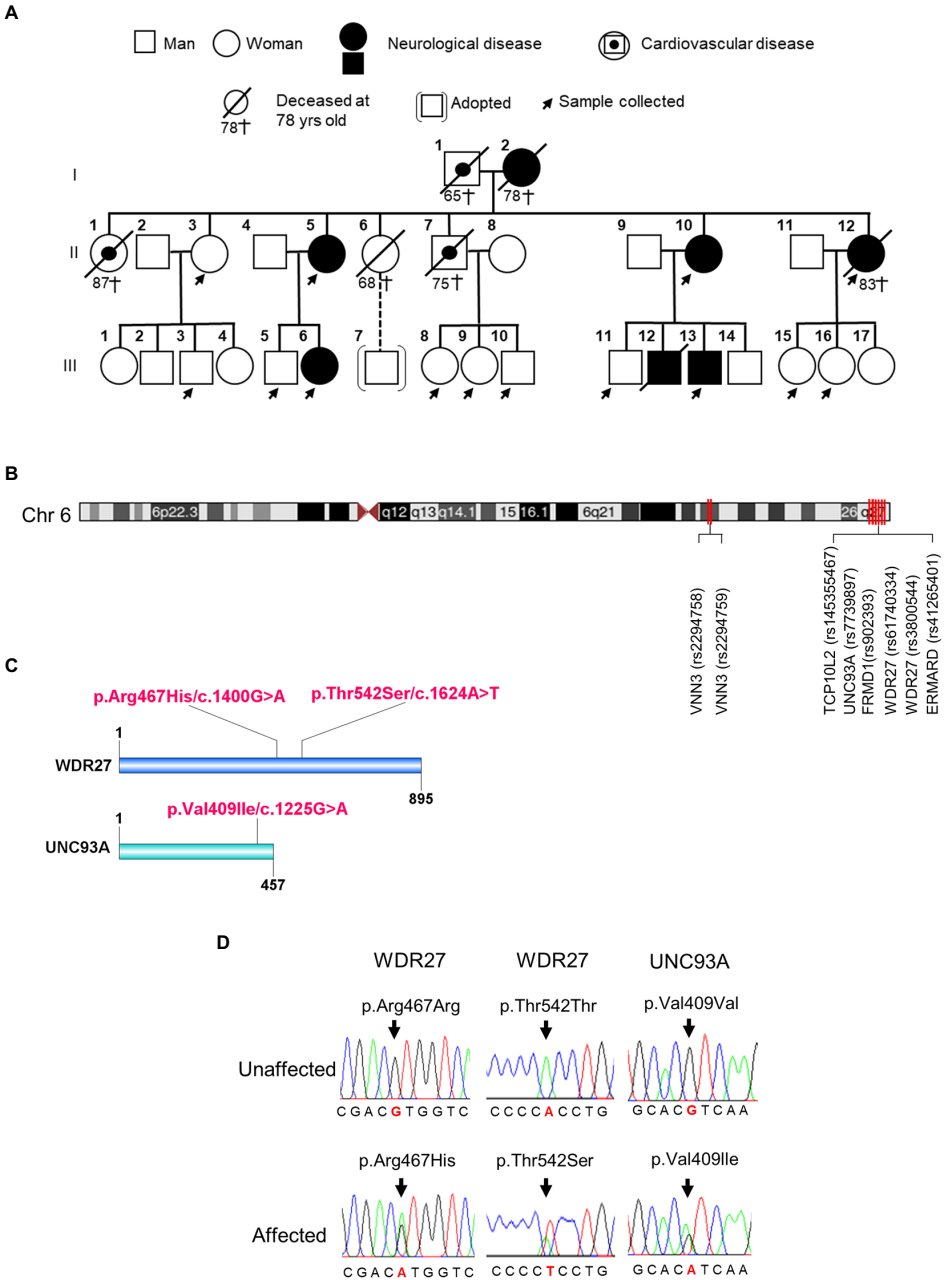
Family pedigrees and variants identified in affected members of family. **(A)** Pedigree of the Peruvian family. **(B)** The chromosomal position of UNC93A and WDR27 genes. **(C)** Location of a mutation in the protein structure. **(D)** An electropherogram from affected probands showing a base pair change in the UNC93A gene (Val409Ile/c.1225G > A) and in the WDR27 gene (Thr542Ser/c.1624A > T; Arg467His/c.1400G > A), compared to healthy controls.

Genealogic investigations allowed us to identify five members of a Peruvian family with a strong clinical history of ADRD, including dementia, Alzheimer’s disease, and schizophrenia across two generations (Figure 1A; Supplementary material Table 1). To detect genetic risk factors associated with the development of the neurologic disorders observed in this family, we performed a WGS analysis on affected and healthy members (*n* = 14) of the family. By using the BGISEQ-500 platform, we obtained an average of 113,895.38 Mb of raw bases. After removing low-quality reads, we obtained an average of 106,676.25 Mb clean reads, identifying a total of 3,933,470 SNPs. We then selected coding variants that met the following two criteria: first, candidate variants that harbored at least one “disruptive” or missense variant, and second, variants that were present in affected probands, but not in unaffected members of the family. As a result, we identified three coding variants that segregated across two generations of affected individuals (Supplementary material Table 2). These variants were found to be located at chr6:167728791 (*UNC93A*; rs7739897), at chr6:170047902 (*WDR27*; rs61740334), and at chr6:170058374 (*WDR27*; rs3800544; Figures 1B,C). Two different Sanger PCR sequencing platforms were used to validate the presence of these SNPs (Figure 1D). Several studies have also found SNPs in genes located on chromosome 6q with a significant connection to neurological diseases (Kohn and Lerer, 2005; Naj et al., 2010); however, the *UNC93A* and *WDR27* variants have not yet been associated with ADRD. It is worthy of note that none of the currently known Alzheimer’s disease-associated variants were present in this Peruvian family.

To further confirm an association between the *UNC93A* and *WDR27* variants and familial genetics risk for neurologic disorders, we analyzed their presence in unrelated healthy Peruvians (*n* = 50) and unrelated individuals with neurological disorders (probable Alzheimer’s disease, *n* = 8). As shown in Supplementary material Table 3, the UNC93A variant (V409I) was present in 1/50 of the healthy group, and the WDR27 variants (Thr542S and Arg467His) were present in 2/50 of the healthy group. Interestingly, the three variants did not co-exist in any healthy individuals and were absent in volunteers diagnosed with probable Alzheimer’s disease with no familial history of ADRD. These findings suggest that the co-occurrence of these three variants may be related to neurological disorders in a Peruvian family.

### 3.2. Structural analysis of the WDR27 and UNC93A variants

**Figure 2:**
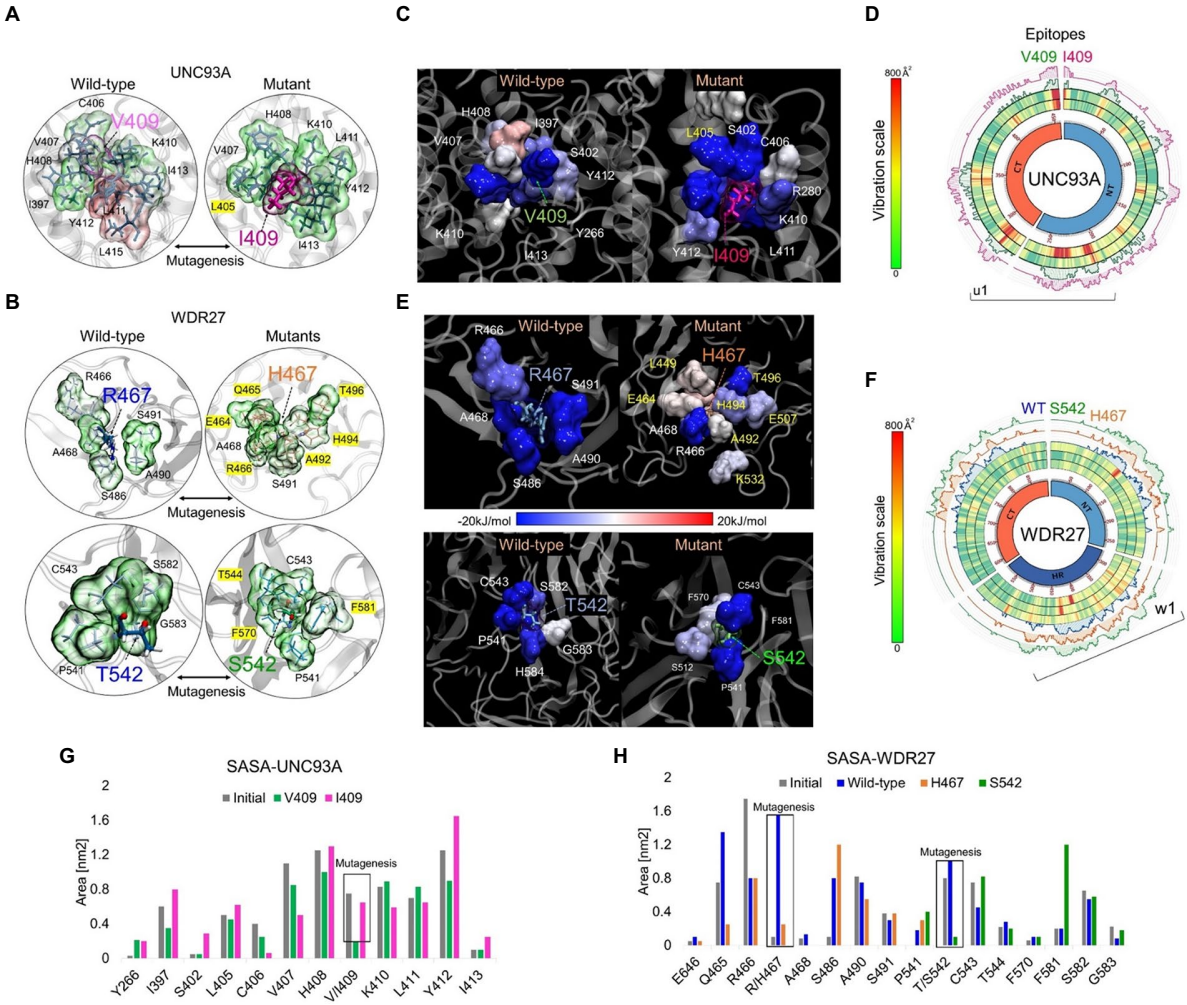
*In silico* analysis of UNC93A and WDR27 amino acid substitutions predict loss of protein function. **(A,B)** show the UNC93A and WDR27 wild-type and mutant proteins**. (C,E)** show the MM-PBSA calculation of the main energetic interactions of residues at mutation sites. Blue indicates favorable energies and red unfavorable energies. **(D,F)** show a Circos plot of the wild-type and mutant full-length protein. The heat map represents the vibrational movement of each residue throughout MD simulations at the scale bar values. The outer histograms show the regions most likely (> 50%) to be epitopes. **(G,H)** show the solvent-accessible surface area (SASA) average values of the wild-type and mutation residues and their neighbors.

UNC93A genes encode a transmembrane protein (457 amino acids) that has 11 alpha-helices and is mainly expressed in the brain, kidney, and liver (Ceder et al., 2017). The WDR27 gene encodes a scaffold protein with multiple WD repeat domains and is ubiquitously expressed in the human body. We used *in silico* approaches to provide insights into the molecular and structural effect of the UNC93A (V409I) and WDR27 (Arg467His and T542S) variants associated with familial ADRD. We, therefore, built the human structure of the UNC93A and WDR27 proteins by homology modeling and performed site-direct mutagenesis to generate the mutated proteins using the UCSF Chimera software (Figures 2A,B). Molecular dynamics simulations (MDS) for 500 ns were performed to stabilize the physical motions of atoms in both proteins to mimic physiological conditions. Importantly, we observed that at the beginning of the MDS (0–50 ns), the residue V409 of the wild-type UNC93 protein interacts with the lipid bilayer of the cell membrane. After 500 ns of MDS, the V409 is internalized toward the protein-active transmembrane conduct region in which the subsequent interactions with ligands or ionic exchanges occur. These movements were characterized by high structural vibration and epitope exposure of the UNC93A’s ectodomain (aa 200–300) of the protein toward the surface of the cell membrane (Figure 2C). In contrast, the mutant I409 blocks the internalization of this residue and loses its capacity to move toward the protein-active transmembrane conduct region due to the loss of about 50% of the global residual vibration (Figure 2D). As a result of this change in the amino acid, the UNC93A loses its capacity for ion exchange and interaction with potential ligands or partners. Regarding the WDR27 protein, both His467 and S542 variants induce the internalization of these residues in the part of the hinge domain of the protein predicting the loss of function and capacity to interact with other partners (Figure 2E). We also observed that the wild-type and mutant proteins are very stable due to their close residual vibration and epitope exposure patterns (Figure 2F). The solvent accessible surface area (SASA) analysis demonstrated that the UNC93A (I409) protein increased the surface area of the mutated amino acid and its environment (Figure 2G), while the WDR27 variants reduced its SASA (Figure 2H). The effect on the amino acid substitutions for both proteins was determined using the Molecular Mechanics Poisson–Boltzmann Surface Area (MM/PBSA) calculation of free energies and energy contribution using the last 150 ns of MD trajectories. Remarkably, the I409 increased the affinity of the protein to the membrane, indicating a reduced capacity for internalization, while the WDR27 variants reduced the protein stabilization of both His467 and S542 mutated amino acids. Together, these findings indicate that the UNC93A and WDR27 variants have a strong effect on the functionality and ability to interact with their environment, and thus potentially affect brain homeostasis.

### 3.3. Functional analysis of loss of WDR27 and UNC93A gene expression *in vitro*

**Figure 3:**
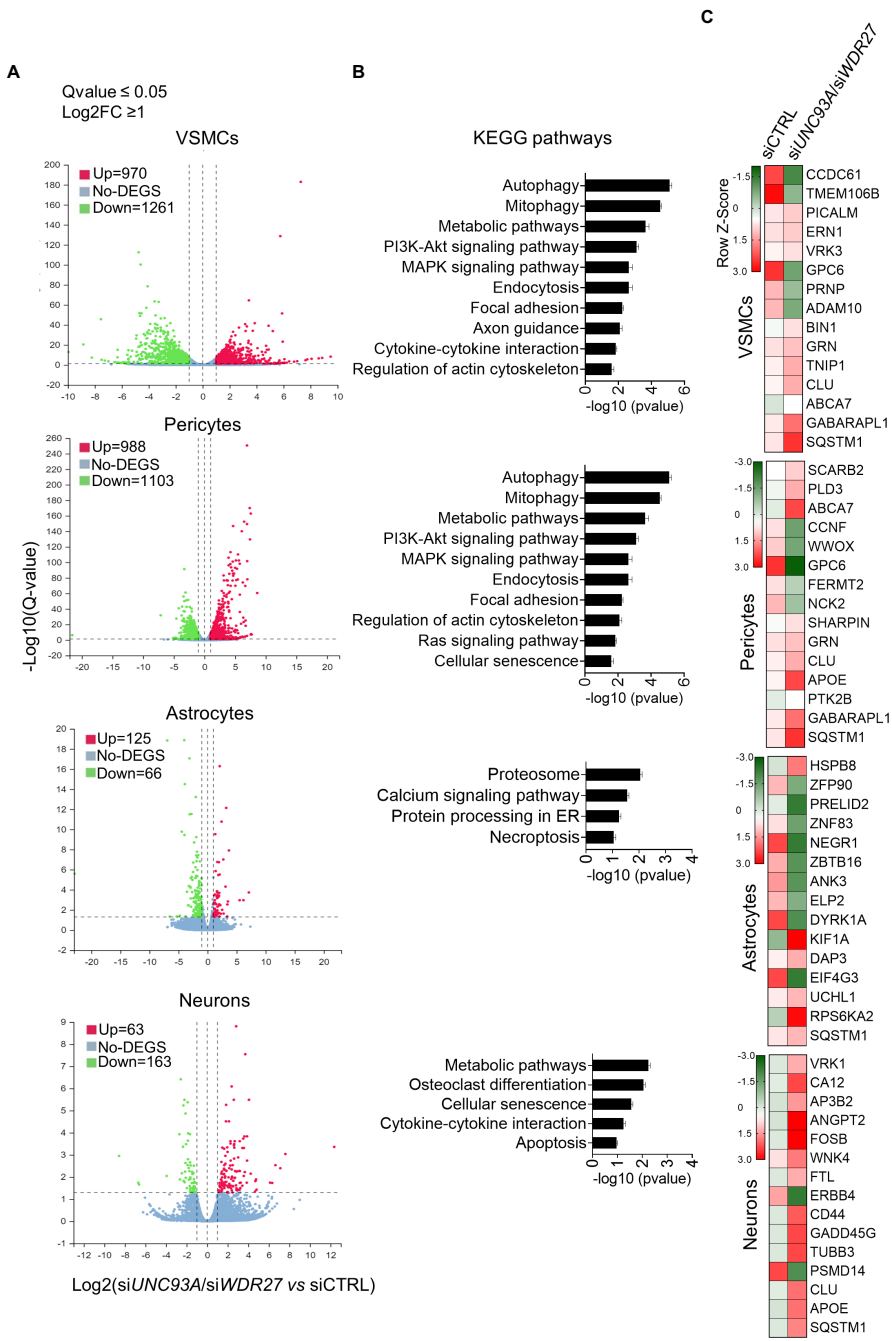
Loss of UNC93A and WDR27 affects the global transcriptomic signature of brain cell lines *in vitro*. **(A)** Volcano plot of dysregulated genes in four different brain cell types. **(B)** Shows the KEGG pathways analysis. **(C)** Heatmap of dysregulated genes previously associated with neurodegenerative disease.

We next investigated how the co-occurring inhibition of *UNC93A* and *WDR27* gene expression affects the cellular homeostasis of brain cell lines, including neurons, pericytes, astrocytes, and VSMCs. To achieve this goal, we simultaneously silenced both *UNC93A* and *WDR27* genes using a siRNA approach and performed a high throughput RNA sequencing (Supplementary material Figure 2). Differential expression analysis demonstrates that VSMCs had the highest numbers of differentially expressed genes (DEG = 2,231), followed by pericytes (DEG = 2091), while astrocytes (DEG = 226) and neurons (DEG = 191) showed a modest change in gene expression compared with control groups (Figure 3A), suggesting that mutations promoting a loss of function of the *UNC93A* and *WDR27* genes affect the neurovascular unit of the brain. Pathway analysis of VSMC and pericyte gene signatures identified enrichment for multiple known molecular pathways associated with neurological disorders, such as impaired autophagy pathways signaling (Li et al., 2016), ubiquitin-mediated proteolysis (Upadhya and Hegde, 2007), unproductive metabolism signaling (Van Der Velpen et al., 2019) inflammation, and necroptosis (Zhang et al., 2021; Figure 3B), while neurons showed a gene signature enriched for cellular senescence, cytokine–cytokine interactions, and apoptosis. Astrocytes showed only modest enrichment for necroptosis and the proteasomal degradation pathway (Figure 3B). Similar to our results, previous *in vivo* studies have reported a direct connection between autophagy activation and UNC93A levels in the healthy brains of mice under starvation, indicating the potential role of UNC93A in metabolic stability, energy uptake, and nutrient transport in the brain (Ceder et al., 2017). Interestingly, several neurological diseases have been associated with defects of autophagy and metabolism homeostasis, including Alzheimer’s, Parkinson’s, and Huntington’s diseases (Wang et al., 2016; Croce and Yamamoto, 2019; Xu et al., 2021). We also observed that multiple genes previously related to Alzheimer’s disease, such as CLU (Jun et al., 2010) SQSTM1 (Cuyvers et al., 2015) GPC6 (Kunkle et al., 2021), and ABCA7 (De Roeck et al., 2019), were dysregulated in brain cells deficient in UNC93A and WDR27 (Figure 3C). Interestingly, the strongest genetic risk factor for Alzheimer’s disease, APOE, was also modulated in pericytes and neurons (Figure 3C).

## 4. Discussion

Recent studies have shown racial disparities in ADRD diagnosis between white and minority groups (Lennon et al., 2022; Suran, 2022). Diverse evidence suggests that there may be racial differences in risk factors associated with the development of ADRD (Mayeda et al., 2017; Brewster et al., 2019; Barnes, 2022). Risk factors such as genetics, age, lifestyle, and co-morbid cardiovascular disease can be useful to understand the incidence, prevalence, and predisposition of an individual to ADRD. Despite the research progress on racial differences in ADRD in developed countries, the diagnosis of ADRD in developing countries (e.g., Asian, African, and South American countries) deserves more recognition for its contribution to the global burden of Alzheimer’s disease (Chávez-Fumagalli et al., 2021). The limited resources to address mental health issues, the lack of adequate technology to diagnose ADRD, and the few funding agencies that support research studies are also major challenges faced by public health systems in developing countries. Indeed, less than 10% of people living with dementia in low-and middle-income countries are diagnosed (Prince et al., 2011). Notably, the Peruvian population has a strong Amerindian ancestral background (approximately 80%), compared to other Latin American populations (Norris et al., 2017, 2020), indicating an opportunity to identify ancestry-specific genetic modifiers associated with the development of ADRD.

Here, we report an inheritance risk factor for ADRD in a Peruvian family with an Amerindian ancestral background. We identified a novel combination of three pathogenic variants in the heterozygous state (*UNC93A*: rs7739897 and *WDR27*: rs61740334; rs3800544) which segregated across two generations in a family with a strong clinical history of ADRD. Notably, the combination of these variants was present in members with neurological disorders but absent in healthy individuals. Importantly, although these three SNPs are fairly common in European American and African American ancestry populations (MAF = 1.78–17.09),^1^ the combined effect of these variants was not previously studied. Our findings thus suggest that the combination of these variants is necessary to manifest the disease. Supporting our hypothesis, it has been reported that variants that have no impact on health when found individually cause severe disease when in combination with other genetic variants (Gifford et al., 2019).

Our *in silico* analysis of the 3D structure of the mutant UNC93A (V409I) and WDR27 (Arg467His and T542S) proteins demonstrates that changes in the amino acid sequences have a dramatic effect on the conformational structure, predicting the loss of function of both proteins. However, the exact biological role of the UNC93A protein remains unknown. For instance, some studies have identified the potential role of UNC93A as a solute carrier and in ion homeostasis (Ceder et al., 2020). Its expression seemed to be associated with increased metabolic activity in organs such as the brain and kidneys (Ceder et al., 2017). In this context, we observed that amino acid substitution (I409) in the UNC93A protein reduced its capacity for ion exchange and interaction with potential ligands or partners, indicating a negative effect on UNC93A bioactivity in the brain. Our *in silico* analysis of the mutant WDR27 proteins demonstrated that both amino acid substitutions induced the internalization of the hinge domain of the protein, affecting its segmental flexibility and ability to clamp down on its substrates or ligands. Similarly, little is known about the WDR27 biological functions in the brain; however, an SNP in the intergenic region adjoining WDR27 (rs924043) was associated with type 1 diabetes (Bradfield et al., 2011), and its duplication has been seen in obese patients (D’Angelo et al., 2018). These results suggest the involvement of UNC93A and WRD27 in metabolic syndrome and related diseases.

Importantly, the brain is the most complex and metabolically active organ, being equipped with a sophisticated network of specialized cell types such as neurons, microglia, astrocytes, pericytes, and VSMCs. In recent years, diverse studies have demonstrated the contribution of these cells to Alzheimer’s disease pathogenesis (Zenaro et al., 2017; Aguilar-Pineda et al., 2021). To investigate the potential effects of a loss of function of UNC93A and WDR27, we used gene silencing technologies to simultaneously reduce the expression of both proteins in four brain cell types to mimic the clinical phenotype of members of the family with ADRD. Our KEGG pathway enrichment analysis showed that autophagy, mitophagy, and metabolic pathways are the most affected in both UNC93A and WDR27 inhibitory conditions. Interestingly, these pathways play an important role in Aβ clearance, and thus dysfunction may lead to the development of Alzheimer’s disease (Zeng et al., 2022). As reduced autophagy activity was related to increased cell death in response to intracellular stress (Galati et al., 2019), these variants could have a negative effect on BBB integrity.

Our study has several limitations. First, a lack of access to imagological studies meant that we were not able to correlate the variants with damaged areas of the brain; however, the MoCA test (Supplementary material Table 1) corroborated that brain areas associated with cognitive domains, predominantly temporal and frontal lobe areas, are damaged. Second, we could not find a validation family for the combination of these variants. However, these combinatory variants may only be present in the reported family. This could be a similar case to that reported for the PSEN1 (E280A) mutation, which only affects the Colombian family descendant of a Spanish conquistador (Lall et al., 2014), or the mutation in the PSEN2 gene (N141I) that is only present in families with German descendants who emigrated to a southern Volga region in Russia in the 1760s (Tomita et al., 1997). Despite these limitations, this study reports for the first time a new genetic risk locus associated with ADRD and the great importance of the UNC93A and WDR27 genes in brain biology.

## Data availability statement

The original contributions presented in the study are publicly available in the Mendeley Data repository. This data can be found here: https://data.mendeley.com/datasets/wsz875f5hs/1, doi: 10.17632/wsz875f5hs.1.

## Ethics statement

The studies involving human participants were reviewed and approved by Comité Institucional de Ética en Investigación Red Asistencial Arequipa—ESSALUD. The patients/participants provided their written informed consent to participate in this study.

## Author contributions

CLLC conceived the work with KLFA and GD-D-C. CLLC and KLFA designed the work. CLLC and KLFA performed the experiments. KLFA, MMO-M, LDG-M, MAC-F, BCC-Q, and KJV-L collected the samples. MMO-M and BCC-Q collected the medical records. JAAP performed the in silico analysis. MFP-C performed and analyzed the neurological tests. PLM analyzed the neurological tests. CLLC and KLFA analyzed and interpreted the data of the study. AP-M provided the clinical samples, and made a substantial contribution to the design of the study. GD-D-C and CLLC supervised the study. CLLC and KLFA wrote the paper. All authors read and approved the final manuscript.

## Funding

This research was funded by the Consejo Nacional de Ciencia, Tecnologia e Innovacion Tecnologica de Peru (grant no 024-2019-Fondecyt-BM-INC.INV). CLLC was supported by NIH (K01HL164687), the MGH Physician–Scientist Development Award, the Ruth L. Kirschstein National Research Service Award (5T32HL007208-43), and the Physician-Scientist Development Award (PSDA-MGH).

## Conflict of interest

The authors declare that the research was conducted in the absence of any commercial or financial relationships that could be construed as a potential conflict of interest.

## Publisher’s note

All claims expressed in this article are solely those of the authors and do not necessarily represent those of their affiliated organizations, or those of the publisher, the editors and the reviewers. Any product that may be evaluated in this article, or claim that may be made by its manufacturer, is not guaranteed or endorsed by the publisher.

## Footnotes

^1^ https://evs.gs.washington.edu/EVS/
