# Supplementary material for: Co-occurring pathogenic variants in 6q27 associated with dementia spectrum disorders in a Peruvian family
Source: Front Mol Neurosci. 2023 Feb 16;16:1104585. doi: 10.3389/fnmol.2023.1104585 (PMC9978490; doi:10.3389/fnmol.2023.1104585)
Supplement: Supplementary file 1 [file Data_Sheet_1.docx]

Supplementary Material

## Supplementary Figures

**
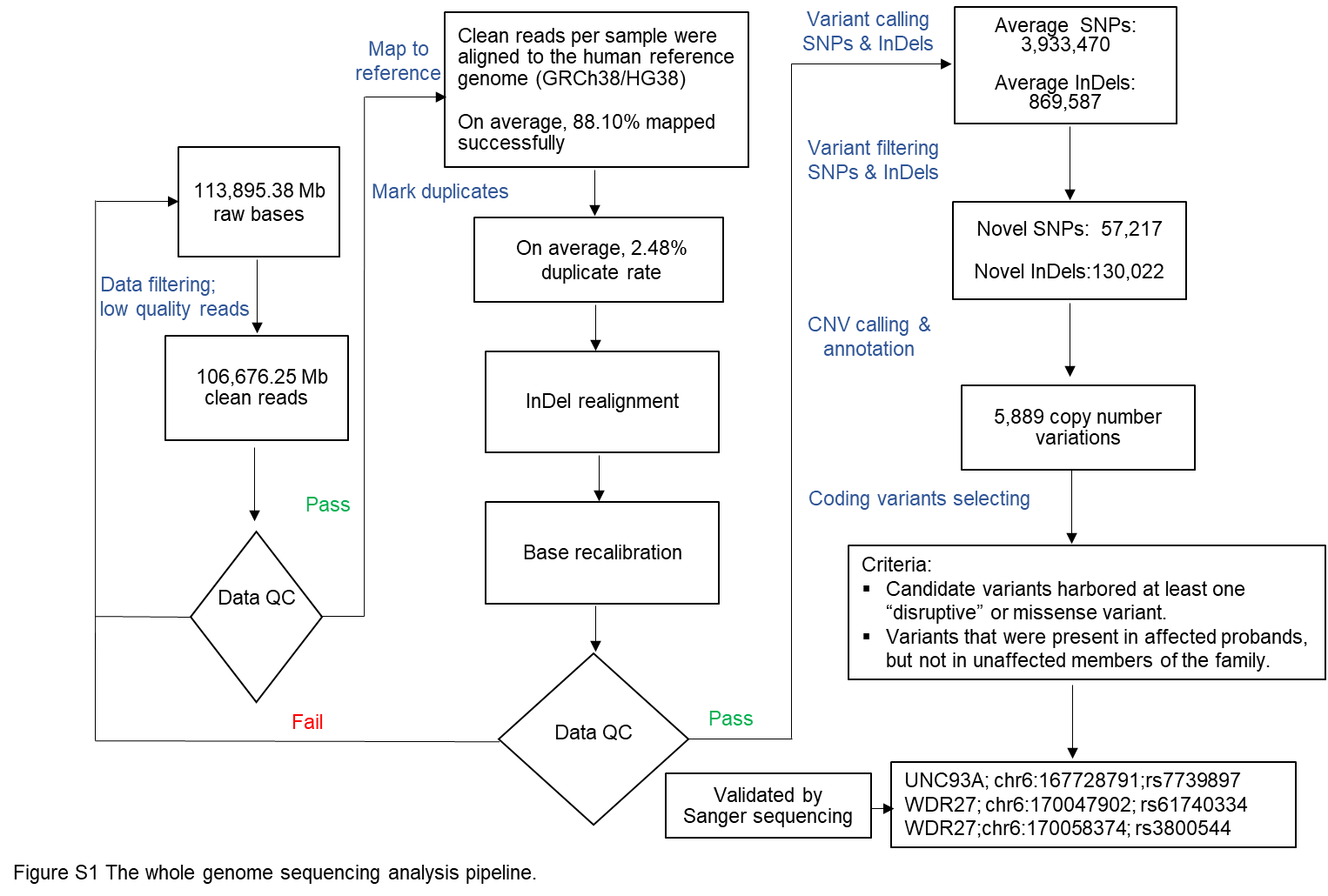
**

**Supplementary Figure 1.** The whole genome sequencing analysis pipeline. QC:quality control.


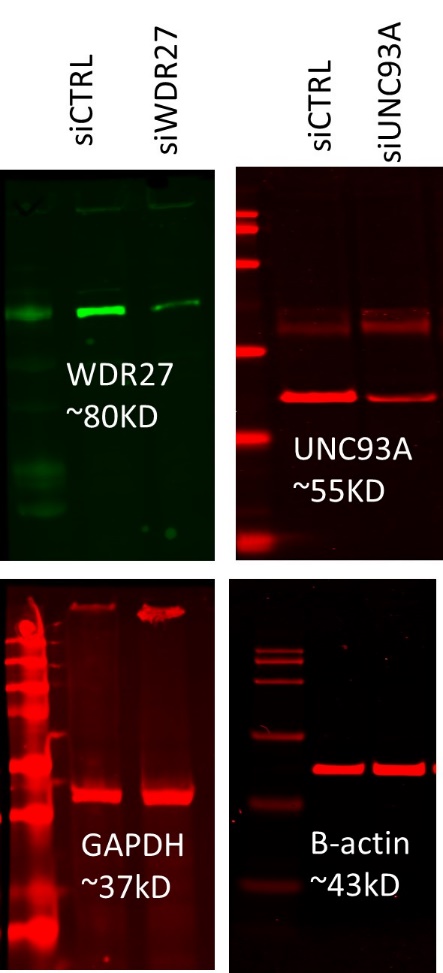


**Supplementary Figure 2.** Western blot results showing gene silencing efficiency of siRNA sequences targeting WDR27 and UNC93A in vascular smooth muscle cells.

# Supplementary Tables

**Supplementary Table 1. Demographic data.**

| Case Nº | Diagnosis | Disease onset (yrs) | Sex | Age (yrs) | Education | MoCa blind test | Clock Drawing Test |
| --- | --- | --- | --- | --- | --- | --- | --- |
| II.3 | Cognitive deficit (age related) | N/A | F | 90 | Basic | 12 | 3 |
| II.5 | ADRD | 79 | F | 89 | Higher | 2 | 0 |
| II.10 | ADRD | N/A | F | 76 | N/A | N/A | N/A |
| II.12 | Alzheimer's disease | N/A | F | 83 | N/A | N/A | N/A |
| III.3 | Normal | - | M | 56 | Higher | 18 | 10 |
| III.5 | Normal | - | M | 49 | Higher | 22 | 7.5 |
| III.6 | Cognitive deficit | Birth | F | 55 | Basic | 12 | 1 |
| III.8 | Normal | - | F | 53 | Higher | 18 | 7 |
| III.9 | Normal | - | F | 51 | Higher | 21 | 10 |
| III.10 | Normal | - | M | 48 | Higher | 20 | 10 |
| III.11 | Normal | - | M | N/A | N/A | N/A | N/A |
| III.13 | Schizophrenia | N/A | M | N/A | N/A | N/A | N/A |
| III.15 | Normal | - | F | N/A | N/A | N/A | N/A |
| III.16 | Normal | - | F | N/A | N/A | N/A | N/A |

MoCa blind test: a score of 18 or above is considered normal.

Clock Drawing Test: a score of 6 or above is considered normal.

N/A: not available.

**Supplementary Table 2. Variants in family members.**

| **Case Nº** | **Diagnosis** | **Age (yrs)** | **Reference SNP number** |
| --- | --- | --- | --- |
| II.3 | Cognitive deficit (age related) | 90 | - |
| II.5 | ADRD | 89 | rs7739897; rs61740334; rs3800544 |
| II.10 | ADRD | 76 | rs7739897; rs61740334; rs3800544 |
| II.12 | Alzheimer's disease | 83 | rs7739897; rs61740334; rs3800544 |
| III.3 | Normal | 56 | - |
| III.5 | Normal | 49 | rs7739897; rs61740334; rs3800544 |
| III.6 | Cognitive deficit | 55 | rs7739897; rs61740334; rs3800544 |
| III.8 | Normal | 53 | - |
| III.9 | Normal | 51 | - |
| III.10 | Normal | 48 | - |
| III.11 | Normal | N/A | - |
| III.13 | Schizophrenia | N/A | rs7739897; rs61740334; rs3800544 |
| III.15 | Normal | N/A | - |
| III.16 | Normal | N/A | - |

SNP: single nucleotide polymorphism; rs: reference sequence

**Supplementary Table 3. Variants in unrelated volunteers.**

| **Gene** | **Variant** | **Reference SNP number** | **Unrelated Controls (n=50)** | **Unrelated volunteers with ADRD (n=8)** |
| --- | --- | --- | --- | --- |
| UNC93A | V409I | rs7739897 | 1 | 0 |
| WDR27 | Thr542S | rs61740334 | 2 | 0 |
| WDR27 | Arg467His | rs3800544 | 2 | 0 |

SNP: single nucleotide polymorphism; rs: reference sequence
